# Ginkgetin Alleviates Inflammation and Senescence by Targeting STING

**DOI:** 10.1002/advs.202407222

**Published:** 2024-11-19

**Authors:** Yadan Liu, Jialin Ye, Zisheng Fan, Xiaolong Wu, Yinghui Zhang, Ruirui Yang, Bing Jiang, Yajie Wang, Min Wu, Jingyi Zhou, Jingyi Meng, Zhiming Ge, Guizhen Zhou, Yuan Zhu, Yichuan Xiao, Mingyue Zheng, Sulin Zhang

**Affiliations:** ^1^ Drug Discovery and Design Center State Key Laboratory of Drug Research Shanghai Institute of Materia Medica Chinese Academy of Sciences Shanghai 201203 China; ^2^ School of Chinese Materia Medica Nanjing University of Chinese Medicine Nanjing 210023 China; ^3^ CAS Key Laboratory of Tissue Microenvironment and Tumor Shanghai Institute of Nutrition and Health Chinese Academy of Sciences Shanghai 200031 China; ^4^ University of Chinese Academy of Sciences Beijing 100049 China; ^5^ Shanghai Institute for Advanced Immunochemical Studies School of Life Science and Technology ShanghaiTech University Shanghai 201210 China; ^6^ Lingang Laboratory Shanghai 200031 China; ^7^ School of Pharmacy East China University of Science and Technology Shanghai 200237 China; ^8^ School of Pharmacology Science and Technology Hangzhou Institute for Advanced Study University of Chinese Academy of Sciences Hangzhou 310024 China

**Keywords:** cGAS‐STING signaling, Ginkgetin, senescence, STING inhibitor, target identification

## Abstract

Ginkgo biloba extract is reported to have therapeutic effects on aging‐related disorders. However, the specific component responsible for this biological function and its mechanism of action remain largely unknown. This study finds that Ginkgetin, an active ingredient of Ginkgo biloba extract, can alleviate cellular senescence and improve pathologies in multiple tissues of aging mice. To reveal the molecular mechanism of Ginkgetin's anti‐aging effect, a graph convolutional network‐based drug “on‐target” pathway prediction algorithm for prediction is employed. The results indicate that the cGAS‐STING pathway may be a potential target for Ginkgetin. Subsequent cell biological and biophysical data confirmed that Ginkgetin directly binds to the carboxy‐terminal domain of STING protein, thereby inhibiting STING activation and signal transduction. Furthermore, in vivo pharmacodynamic data showed that Ginkgetin effectively alleviates systemic inflammation in *Trex1* ^−/−^ mice and inhibits the abnormally activated STING signaling in aging mouse model. In summary, this study, utilizing an artificial intelligence algorithm combined with pharmacological methods, confirms STING serves as a critical target for Ginkgetin in alleviating inflammation and senescence. Importantly, this study elucidates the specific component and molecular mechanism underlying the anti‐aging effect of Ginkgo biloba extract, providing a robust theoretical basis for its therapeutic use.

## Graphical abstract

An artificial intelligence algorithm is utilized to predict and confirm that STING serves as a critical target for Ginkgetin, an active ingredient of Ginkgo biloba extract, combined with pharmacological methods. In vivo experiments indicate that Ginkgetin effectively alleviates systemic inflammation in *Trex1* ^−/−^ mice and inhibits the aging phenotype and the abnormally activated STING signaling in aging mice.

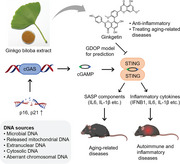

## 1 Introduction

Senescence is a cellular process characterized by permanent growth arrest of damaged or aged cells.^[^ ^1^, ^2^ ^]^ The prevalence of senescent cells rises with the aging process, correlating with the decline in body function and the onset of aging‐related diseases. Oxidative stress, chronic inflammation, and DNA damage are hallmark features of senescent phenotypes.^[^ ^3^, ^4^ ^]^ Specifically, a key characteristic of senescent cells is the secretion of inflammatory mediators, including various cytokines, chemokines, extracellular matrix proteins, and growth factors, collectively referred to as the senescence‐associated secretory phenotype (SASP).^[^ ^5^, ^6^ ^]^ The inflammatory response associated with the SASP is believed to underlie many of aging and aging‐related disorders.^[^ ^7^, ^8^, ^9^ ^]^ Additionally, the activity of the lysosomal enzyme senescence‐associated beta‐galactosidase (SA‐*β*‐gal), a marker for lysosomal activity, is extensively used to assess senescence.^[^ ^4^, ^10^ ^]^ Moreover, senescent cells show increased expression of cyclin‐dependent kinase Inhibitors (CDKis) p16 and p21, which are crucial regulators of cell cycle arrest in senescent eukaryotic cells.^[^ ^4^, ^11^ ^]^

Ginkgo biloba extract has been widely used in clinical settings to treat central nervous system and cardiovascular diseases. Accumulating evidence has revealed that Ginkgo extract possesses anti‐inflammatory^[^ ^12^ ^]^ and anti‐oxidant^[^ ^13^ ^]^ properties and is effective in treating aging‐related diseases.^[^ ^14^, ^15^, ^16^, ^17^, ^18^, ^19^ ^]^ However, the specific component responsible for its anti‐aging activity remains unclear. Ginkgetin, a natural biflavone isolated from Ginkgo biloba leaves,^[^ ^20^ ^]^ has been shown to exhibit numerous bioactivities.^[^ ^21^, ^22^, ^23^, ^24^, ^25^ ^]^ Notably, Ginkgetin exhibits inhibitory activity against oxidation, inflammation, and DNA damage, all of which are major factors inducing senescence.^[^ ^3^, ^4^, ^14^ ^]^ These studies all suggest that Ginkgetin has significant potential for anti‐aging. Interestingly, a recent study^[^ ^26^ ^]^ also identified Ginkgetin as a novel senolytic agent using computational screening, which is consistent with our expectations.

The cyclic GMP‐AMP synthase (cGAS) – stimulator of interferon genes (STING) signaling pathway is the primary mechanism mediating the DNA immune response.^[^ ^27^ ^]^ The cytosolic DNA sensor cyclic cGAS in the cytoplasm can recognize and bind to DNA from viruses, bacteria, and its own cytoplasm, leading to its activation. Upon activation, cGAS synthesizes the second messenger 2’,3’‐cyclic GMP‐AMP (cGAMP), which then binds to the ligand binding domain (LBD) of STING.^[^ ^28^ ^]^ This binding subsequently activates IRF3 and NF‐κB, inducing the expression of type I interferons and proinflammatory cytokines, thus impacting the body's host defense and inflammation responses.^[^ ^29^ ^]^ However, abnormal self‐DNA, such as cytosolic accumulation of nucleic acids due to TREX1 deficiency, or gain‐of‐function mutations in STING (such as STING^N154S^ and STING^V155M^), has been reported to overactivate the cGAS‐STING signaling pathway, leading to autoimmune diseases.^[^ ^29^ ^]^ Moreover, recent breakthroughs have established a key role for the abnormal activation of the cGAS‐STING signaling pathway in cellular senescence.^[^ ^11^, ^30^, ^31^, ^32^ ^]^ For example, diverse senescence stimuli, such as oxidative stress and inflammation, can engage the cGAS‐STING pathway.^[^ ^11^, ^33^, ^34^ ^]^ The cGAS can recognize cytoplasmic chromatin fragments (CCFs) in senescent cells, a hallmark of cellular senescence,^[^ ^35^, ^36^ ^]^ and trigger the production of senescence markers like *p16*, *p21*, and *IL6*, thereby promoting senescence induction. In conclusion, cGAS‐STING pathway plays a significant role in the pathogenesis of cellular inflammation and aging diseases, making it a highly attractive drug target.

In this study, Ginkgetin, a biflavonoid component of Ginkgo biloba extract, was identified as a novel anti‐aging agent. Utilizing predictions from an artificial intelligence model and subsequent biological evaluations, we discovered that Ginkgetin binds to the carboxy‐terminal domain of STING proteins, thereby inhibiting STING activation and signal transduction in vitro and in vivo. Notably, Ginkgetin was found to alleviate the senescence phenotype and inhibit the abnormally activated STING signaling pathway in aging mouse models. These findings provide a strong theoretical basis for using Ginkgetin and Ginkgo biloba extract to treat senescence and inflammatory diseases. The discovery of Ginkgetin's therapeutic target using artificial intelligence algorithms offers a new perspective and paradigm for identifying natural product targets, underscoring the broad potential of artificial intelligence algorithms in drug target discovery.

## 2 Results

### 2.1 Ginkgetin Alleviates Cellular Senescence and Improves Pathologies in Multiple Tissues of Aging Mice

**Figure 1:**
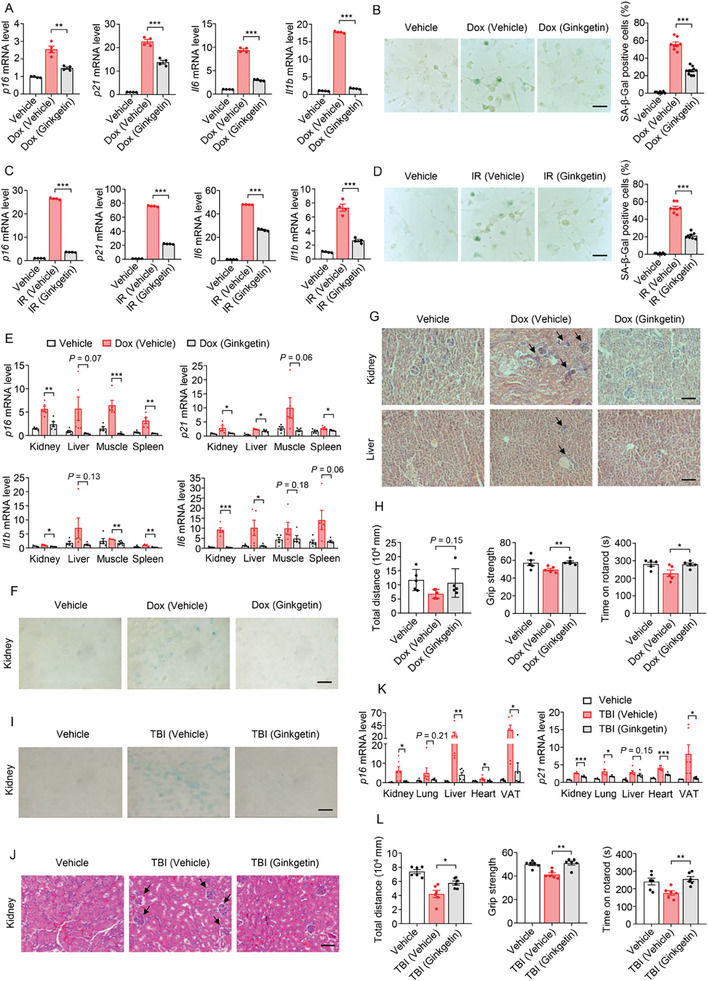
Ginkgetin alleviates cellular senescence and improves pathologies in multiple tissues of aging mice. A,B) MEFs were pretreated with 100 nm Dox for 24 h and subsequently treated with 2 µm Ginkgetin for an additional 48 h. The transcriptional levels of *p16*, *p21*, *Il6*, and *Il1b* mRNA in MEFs were measured by RT‐qPCR (A). SA‐*β*‐Gal staining was also examined and quantified in MEFs, scale bars represent 50 µm (B). C,D) MEFs were irradiated with 6 Gy X‐ray and subsequently treated with 2 µm Ginkgetin for 5 days. The transcriptional levels of *p16*, *p21*, *Il6*, and *Il1b* mRNA in MEFs were measured by RT‐qPCR (C). SA‐*β*‐Gal staining was also examined and quantified in MEFs, scale bars represent 50 µm (D). E‐H) C57BL/6J mice were intraperitoneally injected with 10 mg kg^−1^ Dox on days 1 and 40, followed by intraperitoneal administration of 5 mg kg^−1^ Ginkgetin every 2 days for 2 months (n = 5). The transcriptional levels of *p16*, *p21*, *Il6*, and *Il1b* mRNA in kidney, liver, muscle, and spleen were measured by RT‐qPCR (E). The representative images of SA‐*β*‐Gal staining in kidney were shown, scale bars represent 100 µm (F). The representative images of hematoxylin and eosin (H&E) staining in kidney and liver of Dox‐induced aging mice were shown, scale bars represent 100 µm (G). The physical functions, including the distance run in the OFT, grip strength, and time on the rotarod before falling, were assessed (H). I‐L) C57BL/6J mice were irradiated with a 5 Gy X‐ray for 8 weeks, followed by intraperitoneal administration of 5 mg kg^−1^ Ginkgetin every 2 days for another 8 weeks (n = 6). The representative images of SA‐*β*‐Gal staining in kidney were shown, scale bars represent 100 µm (I). The representative images of H&E staining in kidney were shown, scale bars represent 100 µm (J). The transcriptional levels of *p16* and *p21* mRNA in kidney, lung, liver, heart, and visceral adipose tissue (VAT) were measured by RT‐qPCR (K). The physical functions, including the distance run in the OFT, grip strength, and time on rotarod before falling, were assessed (L). Data are shown as mean ± SEM from at least three independent experiments; a two‐tailed unpaired t‐test was used to analyze significant differences between groups. *P < 0.05; **P < 0.01; ***P < 0.001.

Ginkgo biloba extract has been proven to possess anti‐inflammatory^[^ ^12^ ^]^ and anti‐oxidant^[^ ^13^ ^]^ properties and is used to treat aging‐related diseases.^[^ ^14^, ^15^, ^16^, ^17^, ^18^, ^19^ ^]^ However, the specific component that holds anti‐aging activity remains unknown. Given the anti‐inflammatory and anti‐DNA damage properties of Ginkgetin,^[^ ^21^, ^25^ ^]^ the potential anti‐senescence activity of Ginkgetin in doxorubicin (Dox)‐ or ionizing radiation (IR)‐induced senescent cells^[^ ^37^, ^38^, ^39^ ^]^ was initially investigated. In Dox‐induced mouse embryonic fibroblasts (MEFs) senescent model, Ginkgetin significantly alleviated cellular senescence phenotypes. Specifically, Ginkgetin downregulated the expression of CDKis, including *p16* and *p21*, as well as SASP‐related genes such as *Il6* and *Il1b* (**Figure** **1**A; Figure S1, Supporting Information), and decreased the number of SA‐*β*‐gal positive cells, a well‐recognized senescence marker (Figure 1B).^[^ ^4^, ^10^ ^]^ Similarly, Ginkgetin also exhibited a substantially ameliorative effect on cellular senescence phenotypes induced by IR in MEFs (Figure 1C,D). In conclusion, Ginkgetin effectively alleviated cellular senescence induced by Dox or IR.

Subsequently, to assess whether Ginkgetin could mitigate the aging phenotype in vivo, mouse aging models induced by Dox and total body irradiation (TBI)^[^ ^40^, ^41^ ^]^ were established. In the Dox‐induced aging mouse models, Ginkgetin administration effectively reduced the elevated expression of CDKis (*p16* and *p21*) and SASP‐related cytokines (*Il1b* and *Il6*) in multiple organs, including the kidney, liver, muscle, and spleen (Figure 1E). Furthermore, following the administration of Ginkgetin, the accumulation of SA‐*β*‐Gal positive senescent cells in the kidney of aging mice was diminished (Figure 1F), and the aggregation of immune cells in the kidney and liver was reduced (Figure 1G), implying that severe inflammation in the kidney and liver of aging mice was ameliorated. We also assessed the impact of Ginkgetin on physical parameters of aging mice by measuring total distance in the open field test (OFT), grip strength, and time on rotarod. The results showed that Ginkgetin administration significantly alleviated the decrease in exercise capacity and strength induced by Dox (Figure 1H). In addition, the role of Ginkgetin in the TBI‐induced aging mouse model was also investigated. Consistent with the findings in the Dox‐induced aging model, Ginkgetin administration efficiently alleviated the aging phenotypes, including accumulated senescent cells (Figure 1I), aggregated inflammatory cells (Figure 1J), upregulation of *p16* and *p21* (Figure 1K), and physical dysfunction (Figure 1L). Additionally, C57BL/6J mice were treated with different doses of Ginkgetin for seven consecutive days to assess its potential toxic effects. The results revealed no significant changes in the body and organ weights of the mice (Figure S2, Supporting Information), suggesting that Ginkgetin exhibited no significant toxic effects within the anti‐aging treatment window.

In summary, these results strongly suggest that Ginkgetin has the potential to reduce the burden of senescent cells induced by Dox and IR. Additionally, it appears to postpone physical dysfunction accelerated by the accumulation of senescent cells, indicating that Ginkgetin possesses anti‐aging properties. Nevertheless, the specific target and molecular mechanisms underlying its regulatory effects on anti‐aging remain to be elucidated.

### 2.2 The cGAS‐STING Pathway was Predicted and Confirmed as an “on‐target” Pathway for Ginkgetin

**Figure 2:**
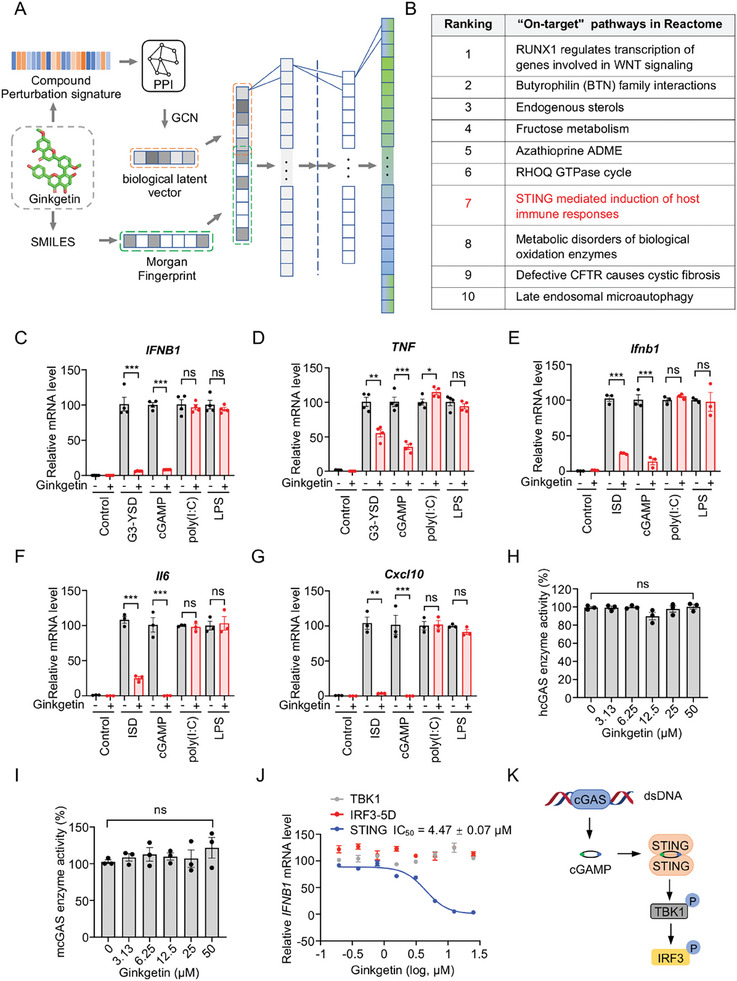
The cGAS‐STING pathway was predicted and confirmed as an “on‐target” pathway for Ginkgetin. A) Ginkgetin‐induced signatures with protein‐protein interactions (PPI) network were passed to a spectral‐based graph convolutional network (sGCN) module to get latent vectors. The SMILES of Ginkgetin was encoded into Morgan fingerprints. Then the latent vectors and Morgan fingerprints were concatenated, and the united vectors were further fed to a deep dense network to predict the “on‐target” pathways. B) The top 10 predicted “on‐target” pathways for Ginkgetin. C,D) THP‐1 mφs were co‐treated with Ginkgetin (10 µm) and various stimuli (G3‐YSD at 1 µg mL^−1^, cGAMP at 5 µm, poly (I:C) at 2.5 µg mL^−1^ and LPS at 10 µg mL^−1^) for 6 h, and the mRNA expression levels of *IFNB1* (C) and *TNF* (D) were measured by RT‐qPCR. E‐G) Raw 264.7 cells were co‐treated with Ginkgetin (10 µm) and various stimuli (ISD at 1 µg mL^−1^, cGAMP at 5 µm, poly (I:C) at 2.5 µg mL^−1^ and LPS at 10 µg mL^−1^) for 6 h, and the mRNA expression levels of *Ifnb1* (E), *Il6* (F) and *Cxcl10* (G) were measured by RT‐qPCR. H,I) Ginkgetin's inhibitory effects on the enzymatic activities of human cGAS (hcGAS) (H) and mouse cGAS (mcGAS) (I) were measured by the PP_i_ase‐coupled cGAS activity assay. J) 293T cells over‐expressing STING, TBK1, or IRF3‐5D were treated with different concentrations of Ginkgetin for 6 h, and the mRNA expression level of *IFNB1* was detected by RT‐qPCR. K) The schematic diagram of the cGAS‐STING pathway. Data are shown as mean ± SEM of at least three independent experiments; a two‐tailed unpaired t‐test was used to analyze significant differences between groups. ns, no statistical difference; *P < 0.05; **P < 0.01; ***P < 0.001.

To elucidate the mechanism of action behind Ginkgetin's anti‐aging activity, a deep learning model, the graph convolutional network‐based drug “on‐target” pathway prediction algorithm (GDOP), was used to predict potential “on‐target” signaling pathways of Ginkgetin. The Top 10 predicted “on‐target” signaling pathways for Ginkgetin, as determined by the GDOP model, are displayed in **Figure** **2**A,B. The complete prediction results were presented in Table S1 (Supporting Information). Notably, the Ginkgetin‐targeted signaling pathways previously reported were ranked high in our model's predictions, such as the Wnt pathway (ranked 1st), oxidation pathway (ranked 8th), and MAPK‐related pathway (ranked 22nd),^[^ ^42^ ^]^ demonstrating our model's predictive accuracy. Additionally, the “STING mediated induction of host immune responses” signaling pathway, ranked 7th, captured our attention. This pathway, primarily initiated by cGAS activation, leads to the production of inflammatory cytokines and type I interferons. Activation of the cGAS‐STING pathway has been previously reported to be associated with cellular senescence, including the production of SASP, cell cycle arrest, and mitochondrial dysfunction.^[^ ^11^, ^38^, ^43^ ^]^ Given Ginkgetin's efficacy in alleviating cellular senescence and improving pathologies in aging mice, we speculated that Ginkgetin may exert its anti‐aging effects by inhibiting the cGAS‐STING signaling pathway.

To confirm our speculation, THP‐1‐derived macrophages (hereafter referred to as “THP‐1 mφs”) and Raw 264.7 cells were stimulated with various immunostimulants, including cGAS agonist (G3‐YSD, ISD), STING agonist cGAMP, RIG‐I/MDA5 agonist polyinosinic‐polycytidylic acid (poly(I:C)), and TLR4 agonist Lipopolysaccharides (LPS). As shown in Figure 2C–G and Figure S3A,B (Supporting Information), Ginkgetin significantly suppressed the upregulation of *IFNB1*, *CXCL10*, *TNF*, and *IL6* induced by cGAS or STING agonist, but not by RIG‐I/MDA5 or TLR4 agonist, indicating that Ginkgetin selectively inhibits the cGAS‐STING signaling pathway. Considering that cGAS is upstream of STING, and that Ginkgetin has a significant inhibitory effect on the agonistic effects of both cGAS and STING agonist, we infer that Ginkgetin targets STING or its downstream components, rather than cGAS. As expected, Ginkgetin has no obvious inhibitory effect on the enzyme activity of human and mouse cGAS (Figure 2H,I). To ascertain whether Ginkgetin specifically targets STING or its downstream components, we over‐expressed STING, TBK1, or IRF3‐5D in 293T cells and explored their impact on *IFNB1* transcription in the presence or absence of Ginkgetin. The results showed that Ginkgetin notably inhibited *IFNB1* mRNA upregulation induced by STING but not by TBK1 or IRF3‐5D (Figure 2J). Given the hierarchical relationship among these signaling components (Figure 2K), these results suggested that STING is a very likely target of Ginkgetin.

### 2.3 Ginkgetin Directly Binds to the Carboxy‐Terminal Domain of STING

**Figure 3:**
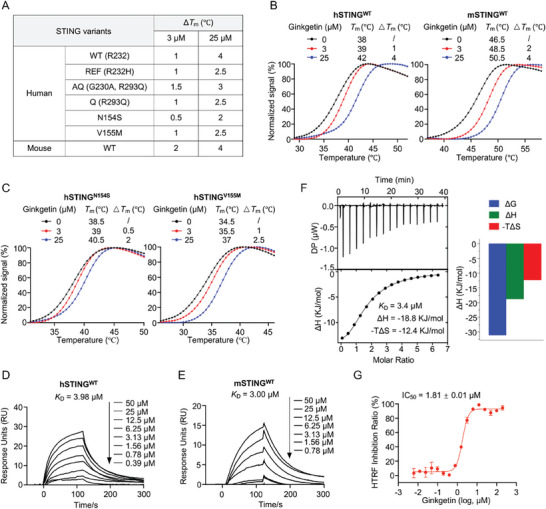
Ginkgetin directly bound to the carboxy‐terminal domain of STING. A) Summary of the effects of Ginkgetin on the thermal stability of various STING proteins. B) The melting curves of hSTING^WT^ and mSTING^WT^ proteins treated with different doses of Ginkgetin in the PTS assay. C) The melting curves of hSTING^N154S^ and hSTING^V155M^ proteins treated with different doses of Ginkgetin in the PTS assay. D,E) The kinetic binding profiles of Ginkgetin with hSTING^WT^ protein (D) and mSTING^WT^ protein (E) analyzed by SPR assay. The *K*_D_ values were determined using a 1:1 kinetics binding model. F) ITC‐binding curves for Ginkgetin titrated into hSTING^WT^. The upper left graph represents the raw ITC thermograms, and the lower left graph represents the fitted binding isotherms. The right graph shows the ΔG, ΔH and ‐TΔS during the period. G) HTRF analysis curve of the competition between Ginkgetin and cGAMP for binding to hSTING^WT^ protein. Data are shown as mean ± SEM from at least three independent experiments.

To confirm whether Ginkgetin directly targets and binds to the STING protein, we purified the carboxy‐terminal domain of mouse STING (mSTING), various human STING alleles (hSTING^R232 (WT)^, hSTING^R232H (REF)^, hSTING^G230A, R293Q (AQ)^, hSTING^R293Q (Q)^), and gain‐of‐function mutant hSTING (hSTING^N154S^, hSTING^V155M^). Binding between Ginkgetin and STING protein was initially assessed using a protein thermal shift (PTS) assay. As summarized in **Figure** **3**A,B and Figure S4A–C (Supporting Information), Ginkgetin effectively and dose‐dependently enhanced the thermal stability of multiple hSTING variants and mSTING proteins. Notably, Ginkgetin also significantly improved the thermal stability of gain‐of‐function mutant hSTING proteins (Figure 3C), suggesting potential inhibitory effects on STING autoactivation mutants. Furthermore, Ginkgetin did not affect the thermal stability of cGAS proteins (Figure S4D,E), indicating there is no interaction between Ginkgetin and cGAS. Subsequently, surface plasmon resonance (SPR) experiments were utilized to assess the kinetic binding properties of Ginkgetin to STING protein. Results revealed that Ginkgetin bound various purified hSTING and mSTING proteins in a slow‐association and slow‐dissociation manner, with *K*_D_ values ranging from 0.5 to 5.0 µm (Figure 3D,E; Figure S4F–I, Supporting Information). To further validate Ginkgetin's binding to STING and exclude the possibility of pan‐assay interference compounds, the isothermal titration calorimetry (ITC) assay, a gold standard assay for determining thermodynamic parameters of target‐ligand interactions, was employed. The results showed that Ginkgetin bound hSTING^WT^ protein exothermically, with a *K*_D_ value of 3.4 µm (Figure 3F). Finally, a homogeneous time‐resolved fluorescence (HTRF) experiment was used to investigate whether Ginkgetin would compete with cGAMP for binding to STING. The results indicated that Ginkgetin competed with cGAMP for binding to hSTING^WT^ with an IC_50_ of 1.81 ± 0.01 µm (Figure 3G). These data collectively demonstrated that Ginkgetin directly bound to the carboxy‐terminal domain of STING.

### 2.4 Ginkgetin Inhibits STING Activation and Signal Transduction

**Figure 4:**
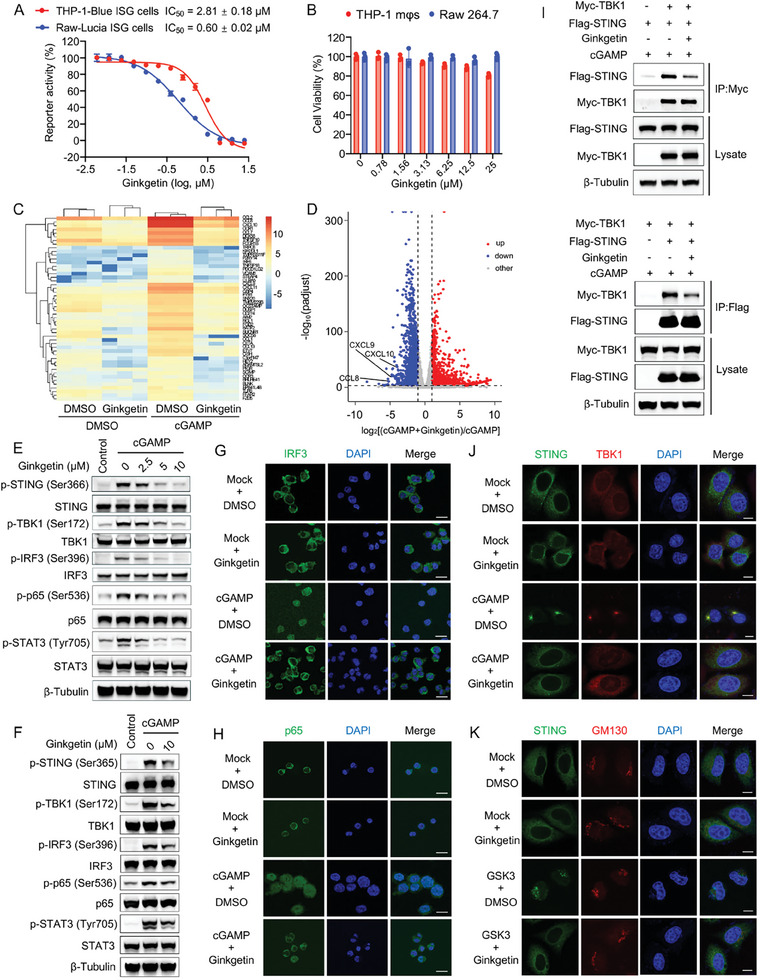
Ginkgetin inhibits STING activation and signal transduction. A) SEAP (Secreted Alkaline Phosphatase) activity in THP‐1 mφs and Luciferase activity in Raw‐Lucia cells treated with Ginkgetin for 24 h in the presence of 5 µm cGAMP. B) Cell viability of THP‐1 mφs and Raw 264.7 cells treated with various concentrations of Ginkgetin for 24 h. C,D) Transcriptomic analysis of THP‐1 mφs treated with indicated compounds for 6 h. cGAMP was used at 5 µm and Ginkgetin at 10 µm. Compared with the cGAMP group, the 50 most significantly downregulated genes in the Ginkgetin and cGAMP co‐treated group are represented by a heatmap (C). Differential genes between the two groups are represented by a volcanic map (D). E,F) Western blot analysis of the phosphorylation levels of key proteins in the cGAS‐STING pathway in THP‐1 mφs (E) and Raw 264.7 (F) cells co‐treated with Ginkgetin and cGAMP for 2 h. G,H) Immunofluorescence imaging for IRF3 (G) or p65 (H) in THP‐1 mφs using confocal microscopy. THP‐1 mφs were pretreated with 10 µm Ginkgetin or DMSO, followed by stimulation with 5 µm cGAMP for 2 h. Scale bars, 20 µm. I) Co‐Immunoprecipitation was performed to detect the interaction between STING and TBK1 using anti‐Myc beads (upper panel) or anti‐Flag beads (lower panel) in 293T cells. The cells were transfected with Flag‐STING and Myc‐TBK1 plasmids for 24 h, followed by 2 h co‐treatment with 10 µm Ginkgetin and 5 µm cGAMP. J,K) Immunofluorescence imaging for STING with TBK1 (J) or STING with GM130 (Golgi autoantigen) (K) in HeLa cells using confocal microscopy. Following transfection with the Flag‐STING plasmid for 24 h, the cells were treated with 10 µm Ginkgetin or DMSO, and subsequently stimulated with 5 µm cGAMP or 1 µm GSK3 for 2 h. Scare bars, 10 µm. Data are presented as mean ± SEM from at least three independent experiments.

To investigate Ginkgetin's inhibitory activity on the STING signaling pathway at the cellular level, reporter gene assays were utilized to evaluate its impact on cGAMP‐induced STING activation. The results indicated that Ginkgetin effectively attenuated the reporter signals induced by STING activation, with an IC_50_ of 2.81 µm in THP1‐Blue ISG cells and a more potent inhibition with an IC_50_ of 0.6 µm in Raw‐Lucia ISG cells (**Figure** **4**A), without obvious toxicity (Figure 4B). Similarly, Ginkgetin exhibited a concentration‐dependent inhibitory effect on double‐stranded DNA (G3‐YSD)‐induced inflammatory cytokine and type I interferon mRNA levels (Figure S5A, Supporting Information). To systematically explore the cell phenotypes associated with Ginkgetin's inhibition of STING, global transcriptomic analysis in THP‐1 mφs was performed. The results indicated that treatment with Ginkgetin alone did not significantly impact the cellular transcription profile. However, the combined use of Ginkgetin and cGAMP significantly inhibited the expression of genes stimulated by cGAMP, including *CCL2*, *CCL8*, *CXCL9*, *CXCL10*, etc (Figure 4C,D). Furthermore, we conducted a differential gene set enrichment analysis (GSEA) comparing the group stimulated with cGAMP alone to the group co‐treated with Ginkgetin and cGAMP. The analysis showed that the three most significantly down‐regulated gene sets were “Interferon gamma response”, “Interferon alpha response”, and “Inflammatory response”, all of which are associated with inflammation response (Figure S5B, Supporting Information). These results suggested that Ginkgetin effectively inhibited the activation of the STING pathway and reduced the production of various inflammatory cytokines.

We then evaluated the impact of Ginkgetin on the phosphorylation levels and translocation of key proteins following the activation of STING signaling pathway. Western blot analysis indicated that treatment with Ginkgetin effectively inhibited the activation of the STING signaling pathway in THP‐1 mφs and Raw 264.7 cells, induced by various STING agonists, such as cGAMP, GSK3, ADU‐S100, and SR‐717. Specifically, the elevated phosphorylation levels of STING and its key downstream proteins, including TBK1, IRF3, p65, and STAT3, were significantly reduced by Ginkgetin treatment (Figure 4E,F; Figure S5C–I, Supporting Information), indicating that Ginkgetin effectively inhibited STING and its downstream signaling pathways. We also employed immunofluorescence (IF) assay to visually observe Ginkgetin's impact on the localization of downstream IRF3 and p65 proteins. As shown in Figure 4G,H, upon activation by cGAMP stimulation, both IRF3 and p65 proteins exhibited pronounced nuclear translocation and aggregation in THP‐1 mφs, whereas they were dispersed in the cytoplasm when co‐treated with Ginkgetin. These results indicate that Ginkgetin efficiently inhibits the activation and nuclear localization of downstream proteins in the STING pathway.

Given that STING is a scaffold protein that recruits downstream signaling proteins when binding to CDNs, we further explored how Ginkgetin affects STING signaling transport. The co‐Immunoprecipitation (Co‐IP) assay showed that Ginkgetin could disrupt the interaction between STING and TBK1 (Figure 4I). Subsequently, we utilized IF assay to investigate the localization of STING protein with TBK1 protein or GM130 protein (a Golgi apparatus marker) in cells. The images revealed that STING agonists promoted perinuclear colocalization of STING and TBK1. However, after treatment with Ginkgetin, both STING and TBK1 were dispersed in the cytoplasm, suggesting the suppression in the translocation of STING and TBK1 (Figure 4J). Moreover, after treatment with Ginkgetin, the localization of STING in the Golgi apparatus induced by the STING agonist GSK3 was reversed, dispersing into the cells, suggesting that the activation and transport of STING was suppressed by Ginkgetin (Figure 4K).

In summary, Ginkgetin affects the translocation of STING from the ER to the Golgi apparatus, thereby weakening the interaction between STING and TBK1, thereby hindering the signal transduction of the STING pathway.

### 2.5 Ginkgetin Suppresses Systemic Inflammation in *Trex1* ^−/−^ Mice

Given Ginkgetin's favorable inhibition and selectivity on the STING pathway in vitro, we next investigated its potential to ameliorate STING‐dependent autoimmune disorders in vivo. TREX1 deficiency occurred in a subset of patients with Aicardi‐Goutières syndrome (AGS) and systemic lupus erythematosus (SLE)^[^ ^44^, ^45^, ^46^ ^]^ and is closely associated with aberrant activation of the cGAS‐STING pathway.^[^ ^38^, ^47^ ^]^ The absence of STING has been shown to alleviate severe and lethal autoinflammatory disease in *Trex1* ^−/−^ mice, highlighting the pivotal role of STING in the pathogenesis of *Trex1*‐deficient diseases.^[^ ^44^ ^]^ Our previous data confirmed that Ginkgetin could directly bind to the CDN binding domain of STING and efficiently inhibit the activation and translocation of STING, leading to suppression of downstream signaling and reduction of inflammatory cytokines. Therefore, the *Trex1* ^−/−^ mouse model was employed to further confirm the inhibitory effect of Ginkgetin on the STING pathway in vivo.

**Figure 5:**
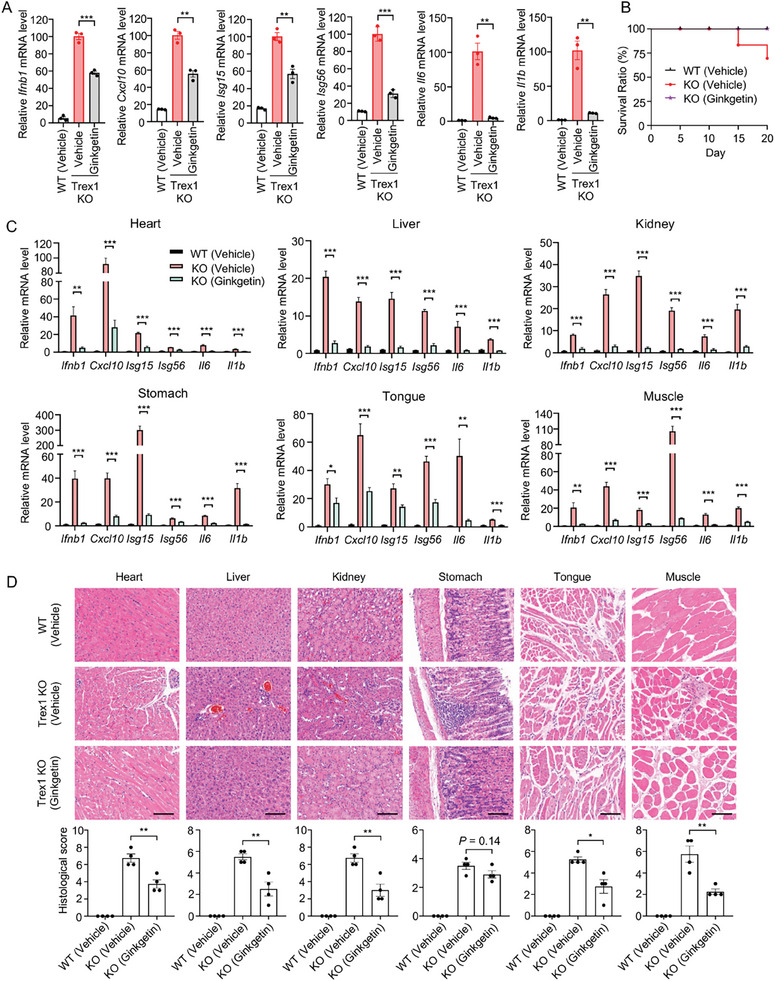
Ginkgetin suppresses systemic inflammation in *Trex1* ^−/−^ mice. A) The mRNA levels of *Ifnb1*, *Cxcl10*, *Isg15*, *Isg56, Il6*, and *Il1b* in BMDMs were detected by RT‐qPCR. BMDMs were derived from *Trex1* ^−/−^ or WT mice and treated with 10 µm Ginkgetin for 24 h. B‐D) The *Trex1* ^−/−^ and WT mice were intraperitoneally treated with Vehicle or 5 mg kg^−1^ Ginkgetin in a solution containing DMSO, PEG400, and 10% hydroxypropyl‐*β*‐cyclodextrin in water (5/5/90, v/v/v), once every 2 days for 20 days (n = 6). At the experimental endpoint, the heart, liver, kidney, stomach, tongue, and muscle were collected and analyzed. Survival curves of mice were shown (B). The mRNA levels for the indicated genes in various organs and tissues were detected by RT‐qPCR (C). Images of H&E staining in various organs and tissues and their blinded histological scores are shown. Scale bars, 200 µm (D). Data are presented as mean ± SEM. A two‐tailed unpaired t‐test was used to analyze significant differences between groups. *P < 0.05, **P < 0.01, ***P < 0.001.

To begin with, we assessed whether Ginkgetin could effectively inhibit STING signaling in bone marrow‐derived macrophages (BMDMs) derived from *Trex1* ^−/−^ mice. As expected, Ginkgetin treatment significantly reduced the expression of *Ifnb1, Cxcl10*, *Isg15*, *Isg56, Il6*, and *Il1b* in *Trex1* ^−/−^ BMDMs (**Figure** **5**A). To evaluate its therapeutic potential in *Trex1* ^−/−^ mice, 6‐8‐week‐old *Trex1* ^−/−^ and wild‐type (WT) mice were intraperitoneally injected with 5 mg kg^−1^ Ginkgetin, once every 2 days for 20 days. During the course of treatment, 2 of 6 untreated *Trex1* ^−/−^ mice died, while none of the 6 mice receiving Ginkgetin treatment died (Figure 5B). At the experimental endpoint, various organs and tissues from surviving mice, including the heart, liver, kidney, stomach, tongue, and muscle, were dissected for RT‐qPCR analysis and H&E staining. The RT‐qPCR results revealed that Ginkgetin significantly inhibited the upregulation of *Ifnb1, Cxcl10*, *Isg15*, *Isg56, Il6*, and *Il1b* in various tissues of *Trex1* ^−/−^ mice (Figure 5C). Furthermore, H&E staining images showed that tissues from *Trex1* ^−/−^ mice exhibited varying degrees of inflammation, as well as infiltration and aggregation of inflammatory cells, which were markedly alleviated by Ginkgetin treatment (Figure 5D). The in vivo data demonstrated that Ginkgetin effectively alleviated systemic inflammation in *Trex1* ^−/−^ mice, indicating its potential to improve STING‐dependent autoimmune diseases.

### 2.6 Ginkgetin Exerts Anti‐Aging Activity by Suppressing cGAS‐STING Pathway

**Figure 6:**
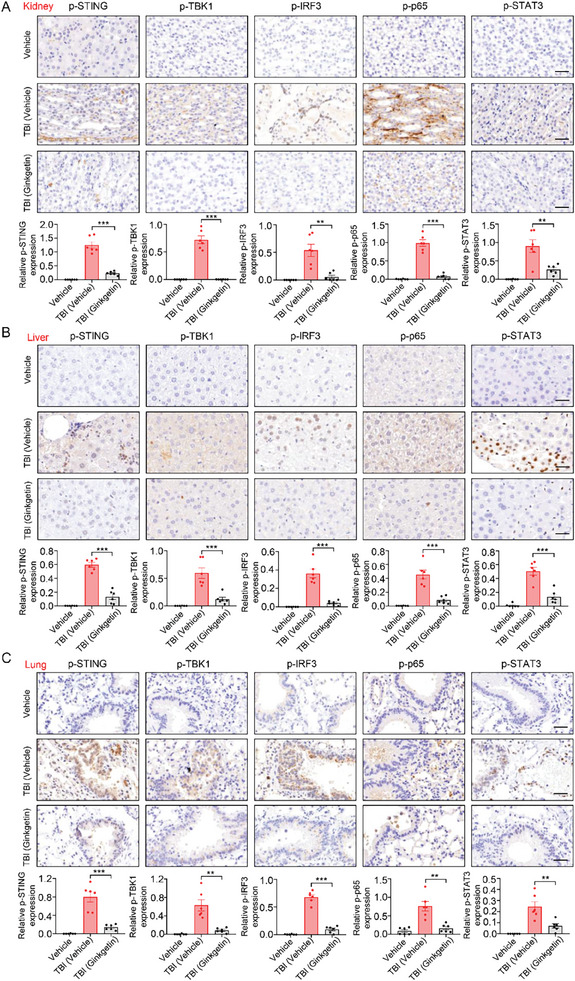
Ginkgetin exerts anti‐aging activity by suppressing the cGAS‐STING pathway. A‐C) The kidney (A), liver (B), and lung (C) tissues were obtained from TBI‐induced aging mouse model and subsequently stained with antibodies against p‐STING (Ser365), p‐TBK1 (Ser172), p‐IRF3 (Ser396), p‐p65 (Ser536), and p‐STAT3 (Tyr705). The representative images were shown, scale bars represent 50 µm. Quantification analysis results were also shown. Data are presented as mean ± SEM. A two‐tailed unpaired t‐test was used to analyze significant differences between groups. **P < 0.01; ***P < 0.001.

Our previous studies have demonstrated that Ginkgetin can effectively alleviate cellular senescence and improve pathologies in multiple tissues of aging mice. We also successfully predicted and confirmed that Ginkgetin inhibits the activation of the cGAS‐STING pathway by targeting STING. Considering that cGAS‐STING pathway serves as a crucial regulator in senescence.^[^ ^11^, ^38^, ^43^ ^]^ It is plausible and essential to investigate whether the anti‐aging activity of Ginkgetin is mediated by inhibiting the activation of the cGAS‐STING pathway. As expected, as shown in **Figure** **6**A–C, the TBI‐induced aging mice exhibited robust activation of STING signaling. Specifically, the phosphorylated levels of key proteins in the cGAS‐STING pathway within kidney, liver, and lung tissues, including STING, TBK1, IRF3, p65, and STAT3, were markedly elevated. More importantly, consistent with the trend that Ginkgetin can effectively alleviate the aging phenotype in aging mice, the abnormally elevated phosphorylation levels of key proteins in the cGAS‐STING pathway were significantly inhibited by Ginkgetin. Overall, these results suggest that Ginkgetin may exert its anti‐aging effect by inhibiting the cGAS‐STING pathway.

## 3 Discussion

In the present study, we found that Ginkgetin, a type of biflavone component from Ginkgo biloba extract, can suppress the expression of senescence markers in senescent cells and aging mouse model induced by doxorubicin or irradiation, as well as alleviate the physical function damage in aging mice. Previous studies have also shown that Ginkgetin can treat aging‐related diseases,^[^ ^22^, ^23^, ^24^ ^]^ and has been identified as a novel senolytic agent,^[^ ^26^ ^]^ but its specific target and molecular mechanism remain unclear. To elucidate Ginkgetin's anti‐aging mechanism, we used a graph convolutional network‐based drug “on‐target” pathway prediction algorithm (GDOP), which takes the structural and biological characteristics of Ginkgetin as input. Using this model, we successfully predicted and verified that Ginkgetin targets the cGAS‐STING signaling pathway. Further biochemical and biophysical data confirmed that Ginkgetin directly binds to the carboxyl terminal domain of STING, thereby inhibiting STING activation. Our research group has used a variety of artificial intelligence algorithms to discover new targets for old drugs with new indications or phenotypes,^[^ ^48^, ^49^ ^]^ demonstrating that artificial intelligence can significantly contribute to drug target identification. This study also provides an experimental basis and a molecular mechanism explanation for the use of Ginkgetin or Ginkgo biloba extract in the treatment of inflammation and aging‐related diseases.

Currently, several agents have emerged as potential treatments for aging‐related diseases, primarily including metformin, NAD+ precursors, GLP‐1 receptor agonists, TORC1 inhibitors, spermidine.^[^ ^50^ ^]^ These agents may also have mitigating effects on other diseases while decelerating or reversing the aging process. Therefore, the therapeutic mechanisms of current anti‐aging drugs are remain inadequately defined. Clarifying the therapeutic mechanisms of anti‐aging interventions and developing new anti‐aging drugs hold significant clinical importance.

Our study indicates that Ginkgetin might slow down the aging process and treat aging‐related diseases by reducing the secretion of SASP and the expression of cell cycle inhibitors (such as *p16* and *p21*). Additionally, we also observed the apparent activation of the STING signaling pathway in the tissues of aging mice, while this activation of the STING signaling was significantly suppressed by Ginkgetin. Taken together, these results indicate that Ginkgetin could alleviate aging and autoinflammatory phenotypes in mice by inhibiting STING activation. Recent studies have also highlighted the critical role of abnormal activation of the cGAS‐STING signaling pathway in cellular senescence,^[^ ^11^, ^30^, ^31^, ^32^ ^]^ consistent with our results.

The cGAS‐STING signaling pathway is an important mechanism for the host to exert an innate immune response, mediating host defense and inflammation. Excessive activation of the STING pathway can lead to inflammatory diseases. In recent years, few types of STING inhibitors have been reported, and individual inhibitors have species specificity. As a STING inhibitor, Ginkgetin not only exhibits strong binding affinity for both human and mouse STING at the molecular level, but also shows effective inhibitory activity on STING activation in human and mouse‐derived cells, indicating that Ginkgetin has no species specificity. Also, it displays promising therapeutic effects on inflammation and autoimmune diseases, such as alleviating inflammatory phenotypes in *Trex1* deficient mice. Moreover, Ginkgetin can interact with gain‐of‐function mutant STING proteins hSTING^N154S^ and hSTING^V155M^, which have been reported to cause spontaneous activation of STING and are associated with STING‐associated vasculopathy with onset in infancy (SAVI).^[^ ^46^, ^51^ ^]^ In future studies, we will further investigate the potential therapeutic effects of Ginkgetin on SAVI and other related diseases, such as Aicardi‐Goutières syndrome (AGS) and systemic lupus erythematosus (SLE). Combining these, we will have a more comprehensive and in‐depth understanding of the function of Ginkgetin. Whether Ginkgetin can be applied to clinical treatment requires further experimental data, including drug safety and biological activity for humans.

## 4 Experimental Section

### Regents

Ginkgetin (purity > 98%) was purchased from MedChemExpress (MCE) (HY‐N0889) and TOPSCIENCE (T4S2126). 2’,3’‐cGAMP (referred to as cGAMP) (HY‐100564A), diABZI STING agonist‐1 (GSK3) (HY‐112921A), ADU‐S100 (HY‐12885A), SR‐717 (HY‐131454), Lipopolysaccharides (LPS) (HY‐D1056) and MSA‐2 (HY‐136927) used in this article were purchased from MCE.

### Cell Lines

The THP1‐Blue ISG (THP‐1) cells were purchased from ATCC and cultured in RPMI 1640 medium with 10% fetal bovine serum (FBS) and 1% penicillin‐streptomycin (PS). MEFs were provided by Dr. Haibing Zhang from Shanghai Institutes for Biological Sciences, Chinese Academy of Sciences; Raw‐Lucia ISG cells (Raw‐Lucia) were purchased from InvivoGen; Raw 264.7 cells were purchased from the Chinese Academy of Sciences Cell Bank; 293T cells and HeLa cells were purchased from Pricella. These cells were cultured in DMEM medium with 10% FBS and 1% PS. THP‐1 derived macrophages (THP‐1 mφs) were induced from THP‐1 cells under the stimulation of phorbol 12‐myristate 13‐acetate (PMA, 100 ng mL^−1^). BMDMs were derived from WT or *Trex1* ^−/−^ C57BL/6J mice as described previously^[^ ^52^, ^53^ ^]^ and cultured in DMEM supplemented with 10% FBS, 1% PS and M‐CSF (20 ng mL^−1^) for 7 days. All cells used in this article were cultured at 37 °C under 5% (v/v) CO_2_ atmosphere. RPMI‐1640 medium (L210KJ) and DMEM medium (L110KJ) were purchased from BasalMedia. FBS was obtained from Gibco (10099141C) and Meilunbio (PWL112). Penicillin‐streptomycin (PWL062) was obtained from Meilunbio.

### Plasmids Transfection

Plasmids used in this article were synthesized by Synbio Technologies. Polyinosinic‐polycytidylic acid (Poly(I:C)) was transfected using Lipofectamine 2000 (Invitrogen, 11668500), while G3‐ended Y‐form Short DNA (G3‐YSD), Interferon Stimulatory DNA (ISD), and plasmids encoding TBK1, IRF3‐5D or STING were transfected with PolyJet (Signagen, SL100688) according to the manufacturer's instructions.

### The Construction of Artificial Intelligence Model, GDOP

1. Model Structure. The compound‐induced signatures with PPI network were passed to a sGCN module to get latent vectors. The SMILES of chemical compounds were encoded into Morgan fingerprints. Then the latent vectors and the Morgan fingerprints were concatenated by the same compound and the united vectors were further fed to a deep dense network to predict the “on‐target” pathways.
2. Data collection. The LINCS phase I L1000 dataset (GSE92742) and updated version were downloaded from the Gene Expression Omnibus (GEO) and LINCS Data Portal 2.0, respectively.^[^ ^54^, ^55^ ^]^ Apart from the signatures, the meta information including targets was curated from the source. The reactome pathways were obtained from the Human Molecular Signatures Database (MSigDB, version 2022.1).^[^ ^56^ ^]^ The human PPI network from the STRING (version 11.5) database was downloaded.
3. Data processing.

Reactome: The large pathways (i.e., over 65 genes) and certain MoA‐unrelated pathways (i.e., CYTOCHROME_P450_ARRANGED_BY_SUBSTRATE_TYPE) were excluded, since large pathways were related to general biological pathways, rather than specific to a certain function.^[^ ^57^ ^]^ Finally, 1001 curated Reactome pathways were obtained.

LINCS: It filtered out the molecules: lack of target information, less five signatures or SMILES couldn't be successfully parsed using rdkit (version 2022.03.5). The profiles for each molecule were averaged by ignoring plate, dose, treatment time and cell line details. The landmark genes were only used. Finally, 2348 valid molecules were kept and randomly split into training (1870), validation (234) and test (234) sets. The positive pathways for each molecule were labelled according to its target information using the curated reactome pathways mentioned above. Like in Zhong et al., for each compound three negative pathways were generated for each positive pathway through a random cross combination of compounds and d pathways.^[^ ^49^ ^]^

STRING: It only kept the nodes present in the “landmark” gene set and the PPI edges with a “combined score” greater than or equal to 800. Accordingly, the curated PPI network consists of 978 nodes and 8320 edges (including self‐connection).

1. The training procedural. Both gene profile and structure features of molecules were applied to train our GDOP model. The profile features were transformed into biological latent vectors by our early developed spectral‐based GCN (sGCN) module in in Zhong etc.^[^ ^49^ ^]^ The sGCN module helps to unify information on the topology of the PPI network and the differential gene expression profiles. The SMILES of chemical compounds were encoded into Morgan fingerprints using rdkit. Then the biological latent vectors and the Morgan fingerprints were concatenated and fed them into a feedforward neural network with four layers of 4096, 3016, 2048, 1024. The dropout rates for each layer, including sGCN layer, were 0.2, 0.5, 0.5, 0.5, 0.2. The rectified linear unit (ReLU) activation function was used for the output of each layer except for the last one. This study used cross entropy as our cost function, and Adam as our optimizer algorithm. Early stopping was used to terminate the training process if the performance of the model on the validation dataset shows no further improvement in specified successive steps.
2. Model evaluation metric. It mainly used the same performance metric, top N accuracy, to evaluate the performance of our model and other methods. This metric reflects the proportion of tested compounds whose any true target can be correctly predicted among the top ranked N targets, and in this study, N values of 100, 30, 10 were evaluated.
3. The implementation of artificial intelligence model, GDOP. The profiles for Ginkgetin were averaged by ignoring plate, dose, treatment time and cell line details. Only the landmark genes and the structure were used as inputs for the pretrained GDOP. At last, the model produced prediction results across 1001 pathways. Codes could be found on https://colinwxl.coding.net/public/sgcn‐otp/sGCN‐OTP/git.

### SEAP and Lucia Reporter Assay

THP1‐Blue ISG (5×10^4 cells) and Raw‐Lucia ISG cells (1×10^4 cells) were seeded into 96‐well plates (NEST) overnight and then co‐treated with gradient concentrations of Ginkgetin and 5 µm cGAMP. After 24 h of treatment, SEAP activity in THP1‐Blue ISG was assessed using the Quanti‐Blue Kit (InvivoGen), and luciferase activity in Raw‐Lucia cells was measured using the Quanti‐Lucia Kit (InvivoGen), following the manufacturer's instructions.

### Cell Proliferation Assay

THP‐1 mφs cells and Raw 264.7 cells were seeded into 96‐well plates separately and incubated in a 37 °C incubator with 5% CO_2_ overnight. The cells were treated with Ginkgetin of gradient concentration for 24 h. The CellTiter‐Glo Luminescent Cell Viability Assay (Promega, G7573) was used to measure cell viabilities according to the manufacturer's instruction.

### Recombinant Protein Expression and Purification

For STING protein expression and purification, the gene sequence encoding C‐terminal domain (residues 139–379) of human STING (hSTING^R232^, hSTING^R232H^, hSTING^S154^, hSTING^M155^, hSTING^R293Q^, hSTING^G230A, R293Q^) and mSTING (residues 139–372) were respectively inserted into the pET28a vector to carry 6×His tag at the N‐terminal. All proteins were expressed in *Escherichia coli* strain BL21 (DE3) (Shanghai Weidi Biotechnology Co., Ltd) cultured in Luria‐Bertani (LB) medium at 37 °C for 4 h and induced with 0.2 mm isopropyl‐*β*‐d‐1‐thiogalactopyranoside (IPTG) (BBI, A600168‐0100) at 18 °C for 16 h. Later, the cells were collected by centrifugation at 3000 rpm for 30 min. The pellets were resuspended and lysed with buffer A (20 mm HEPES (pH 7.4), 200 mm NaCl, 50 mm imidazole, 1 mm Tris (2‐carboxyethyl) phosphine (TCEP)) and sonicated for 15 min, then centrifuged at 16 000 rpm for 60 min. The cell supernatant was flowed through Histrap FF column (GE Healthcare) and the target proteins were eluted by buffer B (20 mm HEPES (pH 7.4), 200 mm NaCl, 1 m imidazole, 1 mm TCEP) in the AKTA system. The purified protein was analyzed by SDS‐PAGE and further purified via Superdex75 10/300GL (GE Healthcare) with system buffer (20 mm HEPES (pH 7.4), 200 mm NaCl). For cGAS protein expression and purification, the gene sequence encoding human cGAS (residues 1–522) and mouse cGAS (residues 1–507) were respectively inserted into the pET28a vector to carry His‐SUMO tag at the N‐terminal. The plasmid was transformed into BL21 (DE3) strain, and protein expression was induced by 0.3 mm IPTG at 18 °C for 16 h. Purification of His‐SUMO cGAS proteins were performed using a standard protocol, as described for STING protein purification.

### Protein Thermal Shift (PTS) Assay

PTS assay was conducted in 96‐well non skirt PCR plate (DN Biotech, 5371012) with a total reaction volume of 20 µL, containing 19 µL of 1.5 µm purified protein and 5 ×SYPRO Orange (Invitrogene, S6651) in PTS buffer (20 mm HEPES (pH 7.4), 200 mm NaCl), and 1 µL of compound. The plate was placed into a real‐time fluorescence quantitative PCR instrument to monitor its fluorescence value from 25 to 90 °C, and the melting temperature (*T*_m_) values were determined using Bio‐Rad software. Then the data were re‐plotted by GraphPad prism 8.0.

### PP_i_ase‐Coupled cGAS Activity Assay

The method of monitoring the enzymatic activity of mcGAS and hcGAS was developed by Zhao etc. with some modifications.^[^ ^58^ ^]^ This experiment was conducted in 384‐well plate (Corning, 3701) with a total reaction volume of 40 µL, including 10 µL of compound, 20 µL of a mixture containing 400 nm cGAS protein, 100 nm *Escherichia coli* pyrophosphatase (Sigma–Aldrich, I5907), 10 µg mL^−1^ HT‐DNA (Sigma, D6898), and 10 µL of a mixture containing 4 mm ATP and 1.2 mm GTP. After a 90‐min incubation at room temperature (RT), 40 µL of 50 mm EDTA (Sigma, 03609) was added to stop the reaction. Finally, 20 µL of malachite green solution (Sigma, 213020) was added for a 30‐min staining, and the absorbance was measured at 620 nm using a microplate reader (TECAN).

### Homogeneous Time‐Resolved Fluorescence (HTRF)

Human STING WT binding kit (Cisbio, 64BDSTGPEG) was used to assess the competitive binding activity of Ginkgetin with cGAMP to hSTING protein. According to the manufacturer's instruction, this experiment was conducted on a white 384‐well plate (Corning, 3600) with a total reaction volume of 20 µL, including 5 µL of Ginkgetin, 5 µL of 6×His‐tagged hSTING protein and 10 µL of a premixture containing d2‐labeled STING ligand (a derivative of the 2'3'‐cGAMP) and 6×His Tb antibody. After incubating 4 h at RT, the HTRF fluorescence value was measured.

### Surface Plasmon Resonance (SPR)

SPR binding assay was conducted on Biacore8K instrument (Cytiva) at 25 °C. Purified STING‐CTD proteins were captured on the CM5 sensor chip. All experiments were conducted in HBS running buffer (10 mm HEPES (pH 7.4), 150 mm NaCl). A series of two‐fold dilutions of Ginkgetin was flowed through the sensor chip at 30 µL min^−1^, with a binding time of 120 s, and a dissociation time of 180 s. The Low Weight Molecular kinetic protocol was used in all binding analysis. The equilibrium dissociation constant (*K*_D_) was determined by fitting the data to a 1:1 binding model using Biacore8K Evaluation software.

### Isothermal Titration Calorimetry (ITC) Binding Assay

The binding parameter of Ginkgetin with hSTING^R232^ protein was measured utilizing MicroCal PEAQ‐ITC calorimeter. All experiments were carried out at 25 °C in the titration buffer with 20 mm HEPES (pH 7.4) and 200 mm NaCl. 5 µm of hSTING^R232^ protein was placed in the sample chamber, Ginkgetin (200 µm) was added using the syringe with twenty successive additions of 2 µL (with an initial injection of 0.4 µL) lasting for 150 s. Data were collected and analyzed using MicroCal PEAQ‐ITC Software.

### Quantitative RT‐PCR

Total RNA was isolated using the RNA‐easy Isolation Reagent (Vazyme, R701‐01) according to the manufacture's instruction, and cDNA was synthesized using the HiScript II Q RT SuperMix for qPCR (Vazyme, R223‐01). Quantitative RT‐PCR was performed in duplicates using ChamQ SYBR qPCR Master Mix (Vazyme, Q311‐02) on a Bio‐Rad or a Quant Studio 5 Flex real‐time PCR system (Applied Biosystems). Human *ACTB* and mouse *Actb* were used as endogenous normalization control to obtained relative expression data. All the primer sequences utilized were shown in Table S2 (Supporting Information). Relative quantification was achieved using the ‐∆∆CT method.

### Western Blotting

The total proteins in the cells were harvested using RIPA lysis buffer (Beyotime, P0013C) supplemented with phosphatase inhibitor (Bimake, B15001) and protease inhibitor (Bimake, B14001). BCA Protein Assay Kit (Thermo Scientific, 23227) was used to determine the concentration of proteins. Equal amounts of samples (30 µg) were separated by 10% SDS‐PAGE and transferred to nitrocellulose (NC) membranes. The membranes were blocked in TBST buffer containing 5% skimmed milk (BD, 232100) at RT for 1 h and then immunoblotted with primary antibodies at 4 °C overnight. The next day, the membranes were subsequently incubated for 2 h at RT with an appropriate secondary antibody and then incubated with ECL luminescence reagent (Meilunbio, MA0186) and visualized using GeneGnome XRQ NPC (Shanghai, China). The antibodies used for western blotting were as followed: anti‐p‐STING (Ser366) (50907S; 1:500), anti‐p‐STING (Ser365) (72971S; 1:500), anti‐STING (13647S; 1:1000), anti‐p‐TBK1 (Ser172) (5483S; 1:500), anti‐TBK1 (3504S; 1:1000), anti‐p‐IRF3 (Ser396) (29047S; 1:200), anti‐IRF3 (11904S; 1:1000), anti‐NF‐κB p‐p65 (Ser536) (3033S; 1:1000), anti‐NF‐κB p65 (8242S; 1:1000), anti‐p‐STAT3 (Tyr705) (9145S; 1:1000), anti‐STAT3 (12640S; 1:1000) and anti‐*β*‐Tubulin (15115S; 1:1000) were purchased from Cell Signaling Technology (CST). Secondary antibody anti‐Rabbit (W4011; 1:10000) was bought from Promega.

### RNA‐Sequencing Experiment

THP‐1 cells were seeded into 10 cm dishes (NEST) and treated with 100 ng mL^−1^ PMA to stimulate macrophage adherence. THP‐1 mφs were incubated with DMSO, 10 µm Ginkgetin, 5 µm cGAMP or co‐incubated with 10 µm Ginkgetin and 5 µm cGAMP for 6 h. Total RNA was extracted with RNA isolater Total RNA Extraction Reagent (Vazyme, R401‐01) after washing with 1×PBS and prepared into RNA library according to the manufacture's instruction from Majorbio RNA‐seq protocol. The subsequent quality inspection, RNA sequencing and differential gene expression analysis of the samples were completed by Majorbio. The heat map and volcano map of the normalized value were drawn by Rstudio and Adobe Illustrator 2021, and the results were obtained by the analysis of differential gene function enrichment using GSEA software.

### Co‐Immunoprecipitation (Co‐IP) Assay

Flag‐STING and Myc‐TBK1 plasmids were transfected into 293T cells by PolyJet for 24 h according to the manufacture's instruction. Then cells were incubated with DMSO, 5 µm cGAMP or co‐incubated with 10 µm Ginkgetin and 5 µm cGAMP for 2 h. Cell pellets were collected and resuspended in Western and IP lysate (Beyotime, P0013) supplemented with phosphatase inhibitor and protease inhibitor. BCA Protein Assay Kit was used to determine the concentration of protein. The lysate was divided into two parts, one for lysate, the other was incubated with anti‐Myc beads (Bimake, B26302) or anti‐Flag beads (Bimake, B26102) for 2 h at RT. The beads were washed three times with the washing buffer (NaCl 136.89 mm; KCl 2.67 mm; Na_2_HPO_4_ 8.1 mm; KH_2_PO_4_ 1.76 mm; 0.5% Tween20) and denaturalized with SDS‐loading buffer by boiling for 5 min. Then the immunoprecipitation and lysate samples were subjected to western blotting analysis. The antibodies used for Co‐IP assay were as followed: anti‐Myc Tag (2278S; 1:1000), anti‐DYKDDDDK Tag (anti‐Flag Tag) (14793S; 1:1000) and anti‐*β*‐Tubulin (15115S; 1:1000) were purchased from CST.

### Immunofluorescence (IF) and Confocal Microscopy

THP‐1 mφs and HeLa cells (transfected with Flag‐STING plasmid for 24 h) grown on coverslips (ThermoFisher, 154534) were incubated with 10 µm Ginkgetin and 5 µm cGAMP or 1 µm GSK3 for 2 h. Then they were fixed with 4% paraformaldehyde fixed solution (Beyotime, P0099) for 30 min, permeabilized in Triton X‐100 (Beyotime, P0096) for 30 min and blocked with 5% bovine serum albumin (BSA) (Sigma, A1933) for 30 min. Cells were stained with indicated primary antibodies at 4 °C overnight and fluorescent‐conjugated secondary antibody for 45 min at RT. The nuclei were counterstained with anti‐fluorescence quenching sealing solution (including DAPI) (Beyotime, P0131). Images were captured using a confocal microscope (Leica). The antibodies used for IF were as followed: anti‐IRF3 (CST, 11904S; 1:200), anti‐NF‐κB p65 (CST, 8242S; 1:400), anti‐STING (Invitrogen, MA5‐26030; 1:200), anti‐TBK1 (Abcam, ab40676; 1:800), anti‐GM130 antibody [EP892Y]‐cis‐Golgi Marker (Abcam, ab52649; 1:200), Alexa Flour 488 Goat anti‐Mouse IgG (Invitrogen, A11029; 1:500), Alexa Flour 488 Goat anti‐Rabbit SFX Kit (Invitrogen, A11034; 1:500), Alexa Flour 633 Goat anti‐Rabbit IgG (H+L) (Invitrogen, A21071; 1:500).

### Animals

All procedures performed on animals followed the guidelines of the Institutional Animal Care and Use Committees (IACUC) of the Shanghai Institute of Materia Medica, Chinese Academy of Sciences (IACUC Issue NO. 2023‐02‐JHL‐31 for C57/BL6J mice). Wild‐type (WT), *Trex1* ^+/−^ and *Trex1* ^−/−^ C57/BL6J mice were used in the experiments. WT C57/BL6J mice were purchased from Beijing Huafukang Biotechnology Co., Ltd. *Trex1* ^+/−^ mice were provided by Xiao Yichuan's group from Shanghai Institute of Nutrition and Health, Chinese Academy of Sciences. *Trex1* ^−/−^ mice were mainly obtained by further mating of male and female *Trex1* ^+/−^ mice and genotyped by standard PCR (primers *Oam878* and *Oge514*). The solvent used for injection was solution containing DMSO, PEG400 and 10% hydroxypropyl‐*β*‐cyclodextrin (HP‐*β*‐CD) in water (5/5/90, v/v/v).

### Acute Toxicity Experiment

WT C57/BL6J mice were randomly divided into four groups (n = 4): Control (0 mg kg^−1^), low dose group (5 mg kg^−1^), medium dose group (10 mg kg^−1^), and high dose group (20 mg kg^−1^). Mice were intraperitoneally injected with different doses of Ginkgetin or Vehicle and weighed daily for one week.

### Aging Mouse Model Experiment

In Dox‐induced aging mouse model experiment, 4‐month‐old male WT C57/BL6J mice received intraperitoneal injections of 10 mg kg^−1^ Dox or Vehicle on day 0 and day 40. Additionally, they were intraperitoneally injected with 5 mg kg^−1^ Ginkgetin or Vehicle once every 2 days for 2 months. In TBI‐induced aging mouse model experiment, 8‐week‐old WT mice were exposed to 5 Gy X‐ray irradiation for 8 weeks. Additionally, they were intraperitoneally injected with 5 mg kg^−1^ Ginkgetin or Vehicle once every 2 days for 8 weeks. The physical function tests were performed at 2 days after the last dose of Ginkgetin. Mouse tissues were collected for RNA extraction, paraffin embedded for H&E staining and IHC staining or frozen in OCT solution for SA‐*β*‐Gal staining. The primer sequences utilized were shown in Table S2 (Supporting Information), marked with *.

### Trex1^−/−^ Mouse Model Experiment

The 6‐8‐week‐old *Trex1* ^−/−^ male and female mice were randomly divided into two groups: KO (Vehicle) and KO (5 mg kg^−1^ Ginkgetin) (n = 6). To assess the in vivo inhibitory effect of Ginkgetin, WT or *Trex1* ^−/−^ mice were injected intraperitoneally with Ginkgetin or Vehicle once every 2 days for 20 days. After euthanasia of mice, the heart, liver, kidney, stomach, tongue and muscle of mice were extracted for H&E staining and RT‐qPCR.

### Histological Analyses

The tissue samples of WT mice and *Trex1* ^−/−^ mice were fixed in 4% paraformaldehyde and sent to Shanghai Ruiyu Biotechnology Co., Ltd. for H&E staining and IHC staining, and the images were viewed and captured by K‐Viewer; IHC results were statistically processed using ImageJ software.

### Senescence‐Associated *β*‐Galactosidase (SA‐*β*‐Gal) Staining for Tissues

SA‐*β*‐Gal staining kit (Beyotime) was used to perform SA‐*β*‐Gal staining according to the manufacturer's instruction. In brief, frozen sections were dried at 37 °C for 20–30 min and fixed for 15 min at RT. Frozen sections were washed three times with PBS and incubated with SA‐*β*‐Gal staining overnight. Samples were examined under a bright‐field microscope and each sample was imaged over 8–10 regions.

### Physical Function Assessment

All physical tests were performed 2 days after the last injection. To evaluate the exercise capacity, mice were placed in an open field test (OFT) and calculated the distance traveled in 20 min. For grip strength measurement, forelimb grip strength was determined using a Grip Strength Meter, with results averaged over 15 trials. Motor coordination ability was measured using an accelerating RotaRod system, which was accelerated from 4 to 40 rpm min^−1^ over a 5 min interval. The duration time was recorded when the mouse dropped from the RotaRod, with the results averaged from four trials.

### Enzyme Linked Immunosorbent Assay (ELISA)

Concentrations of the cytokines in serum of mice were measured by ELISA Kit according to the manufacturer's introductions. Mouse IL‐6 ELISA Kit (abs520004‐96T) and Mouse IL‐1 beta ELISA Kit (abs520001‐96T) were purchased from Absin. Mouse IFN‐*β* ELISA Kit was purchased from R&D SYSTEMS (DY8234‐05).

### Statistical Analysis

All data were shown as mean ± SEM from at least three independent experiments. Two‐tailed unpaired t‐test was used for statistical analysis with Graphpad Prism Software. Statistical significance was denoted in the figures as follows: ns, no statistical difference, *P* > 0.05; *P < 0.05; **P < 0.01; ***P < 0.001.

## Conflict of Interest

The authors declare no conflict of interest.

## Author Contributions

Y.L., J.Y., Z.F., X.W., and Y.Z. contributed equally to this work. S.Z. and M.Z. performed conception of the hypothesis. S.Z., M.Z., and Y.X. performed study supervision. Y. L, J.Y., Z.F., and X.W. performed development of methodology. Y.L., J.Y., Z.F., X.W., R.Y., Y.Z. performed acquisition of data. Y.L., J.Y., Z.F., X.W., R.Y., Y.Z., B.J., Y.W., J.Z., J.M., Z.G., G.Z., and Y.Z. performed analysis and interpretation of data. Y.L., J.Y., Z.F., X.W., S.Z., and M.Z. wrote, review, and/or revision of the manuscript. All authors discuss the study. The manuscript was written through contributions of all authors. All authors have given approval to the final version of the manuscript.

## Supporting information

Supporting Information

Supplemental Table 1

Supplemental Table 2

## Data Availability

The data that support the findings of this study are available from the corresponding author upon reasonable request.
