# Supplementary material for: Ginkgetin Alleviates Inflammation and Senescence by Targeting STING
Source: Adv Sci (Weinh). 2024 Nov 19;12(2):2407222. doi: 10.1002/advs.202407222 (PMC11727237; doi:10.1002/advs.202407222)
Supplement: Supplementary file 1 — Supporting Information [file ADVS-12-2407222-s003.pdf]

ADVANCED SCIENCE
Open Access

Supporting Information

for *Adv. Sci.*, DOI 10.1002/advs.202407222

Ginkgetin Alleviates Inflammation and Senescence by Targeting STING

Yadan Liu, Jialin Ye, Zisheng Fan, Xiaolong Wu, Yinghui Zhang, Ruirui Yang, Bing Jiang, Yajie Wang, Min Wu, Jingyi Zhou, Jingyi Meng, Zhiming Ge, Guizhen Zhou, Yuan Zhu, Yichuan Xiao, Mingyue Zheng* and Sulin Zhang**

Ginkgetin Alleviates Inflammation and Senescence by Targeting STING

Yadan Liu, Jialin Ye, Zisheng Fan, Xiaolong Wu, Yinghui Zhang, Ruirui Yang, Bing Jiang, Yajie Wang, Min Wu, Jingyi Zhou, Jingyi Meng, Zhiming Ge, Guizhen Zhou, Yuan Zhu, Yichuan Xiao, Mingyue Zheng*, Sulin Zhang**

Y. Liu, X. Wu, Y. Zhang, R. Yang, B. Jiang, Y. Wang, M. Wu, J. Zhou, J. Meng, Z. Ge, G. Zhou, Y. Zhu, M. Zheng, S. Zhang

Drug Discovery and Design Center

State Key Laboratory of Drug Research

Shanghai Institute of Materia Medica

Chinese Academy of Sciences

Shanghai 201203, China

E-mail: myzheng@simm.ac.cn (M. Zheng)

slzhang@simm.ac.cn (S. Zhang)

J. Ye, Y. Xiao

CAS Key Laboratory of Tissue Microenvironment and Tumor

Shanghai Institute of Nutrition and Health

Chinese Academy of Sciences

Shanghai 200031, China

E-mail: ycxiao@sibs.ac.cn

J. Ye, Y. Zhang, R. Yang, Y. Xiao, M. Zheng, S. Zhang

University of Chinese Academy of Sciences

Beijing 100049, China

X. Wu

School of Pharmacy

East China University of Science and Technology

Shanghai 200237, China

Y. Liu, B. Jiang, J. Meng, Y. Zhu, M. Zheng

School of Chinese Materia Medica

Nanjing University of Chinese Medicine

Nanjing 210023, China

Z. Fan, G. Zhou, M. Zheng

Shanghai Institute for Advanced Immunochemical Studies

School of Life Science and Technology

ShanghaiTech University

Shanghai 201210, China

Y. Wang, Z. Ge, M. Zheng

School of Pharmacology Science and Technology

Hangzhou Institute for Advanced Study

University of Chinese Academy of Sciences

Hangzhou 310024, China

Z. Fan, M. Zheng

Lingang Laboratory

Shanghai 200031, China

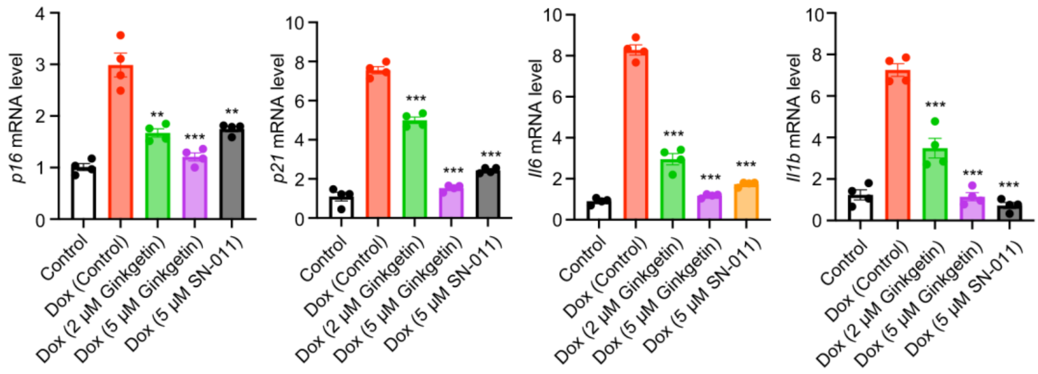

Figure S1. The alleviating effect of Ginkgetin on the MEFs aging model induced by Dox. MEFs were pretreated with 100 nM Dox for 24 h and subsequently treated with Ginkgetin or SN-011 for an additional 48 h. The transcriptional levels of *p16*, *p21*, *Il6,* and *Il1b* in MEFs were measured by RT-qPCR. Data are shown as mean ± SEM from four independent experiments; a two-tailed unpaired t-test was used to analyze significant differences between groups. ***P* < 0.01; ****P* < 0.001.

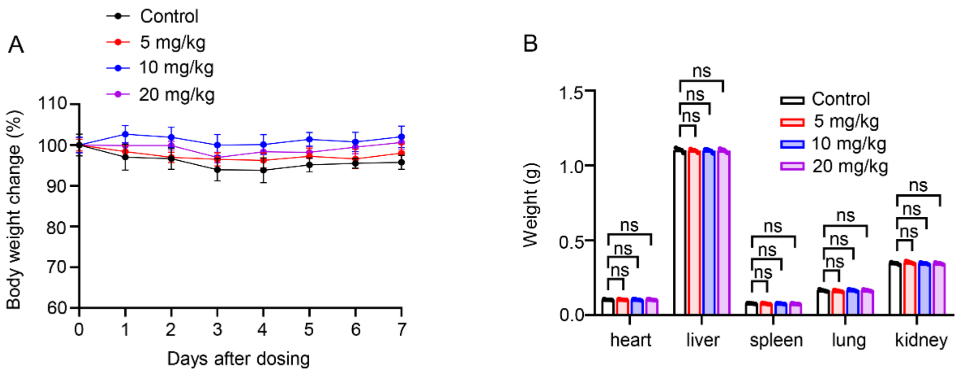

Figure S2. Ginkgetin exhibited no toxicity in mice within the experimental dose range. (A) Body weight changes of mice during the acute toxicity experiment (n=5). (B) Organ weight statistics of mice (n=5). Data are presented as mean ± SEM; a two-tailed unpaired t-test was used to analyze significant differences between groups. ns, no statistical difference.

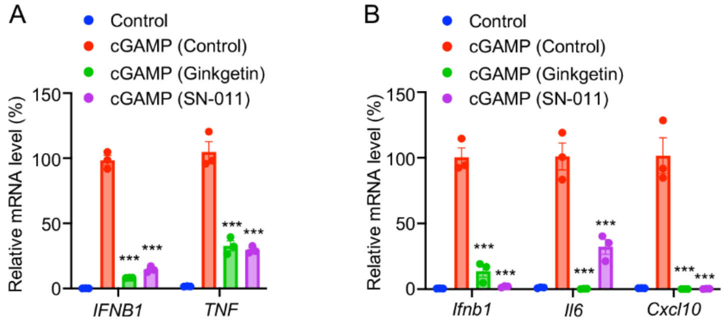

Figure S3. The inhibitory effect of Ginkgetin on the STING signaling pathway. (A) THP-1 mφs were co-treated with cGAMP (5 μM) and Ginkgetin (10 μM) or SN-011 (10 μM) for 6 h, and the mRNA expression levels of *IFNB1* and *TNF* were measured by RT-qPCR. (B) Raw 264.7 cells were co-treated with cGAMP (5 μM) and Ginkgetin (10 μM) or SN-011 (10 μM) for 6 h, and the mRNA expression levels of *Ifnb1*, *Il6* and *Cxcl10* were measured by RT-qPCR. Data are shown as mean ± SEM from three independent experiments; a two-tailed unpaired t-test was used to analyze significant differences between groups. ***P < 0.001.

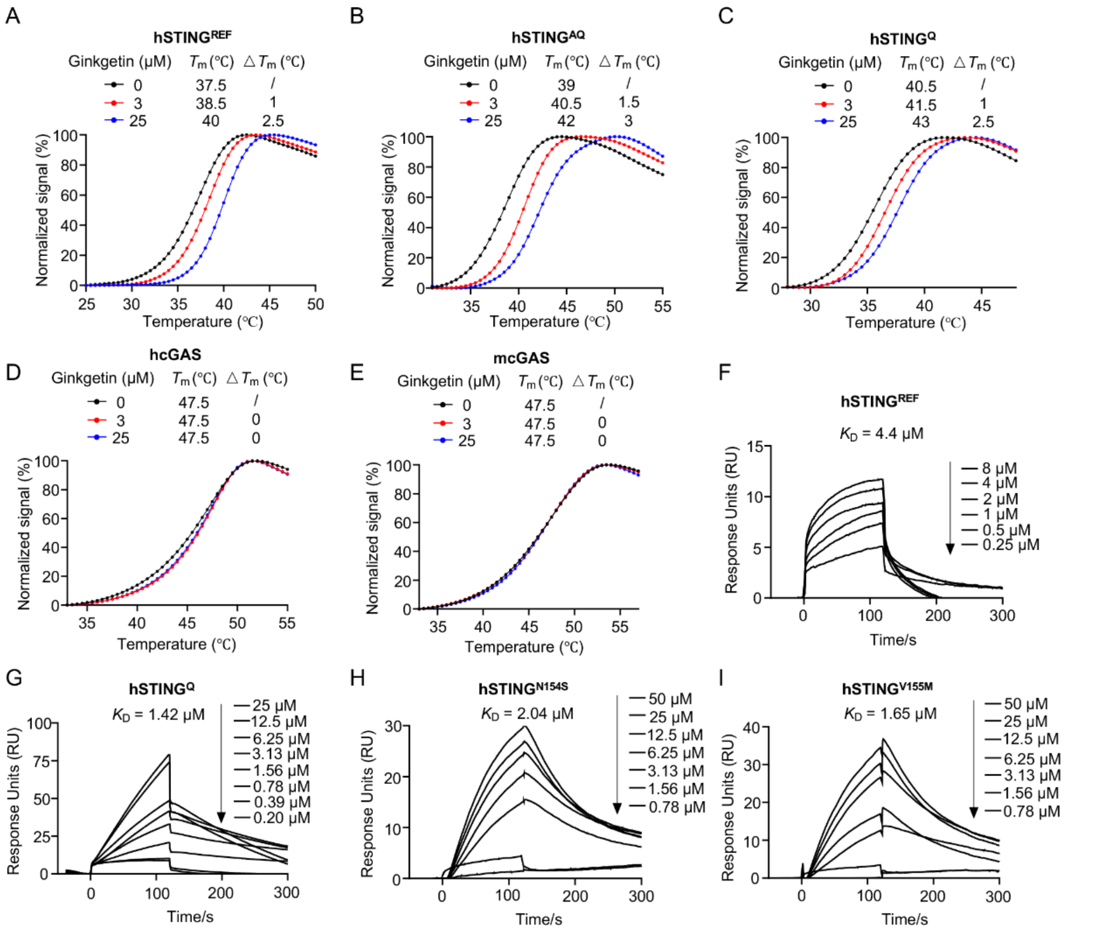

Figure S4. Ginkgetin directly binds to the carboxy-terminal domain of STING. (A-C) The melting curves of $hSTING^{REF}$ (A), $hSTING^{AQ}$ (B), and $hSTING^{Q}$ (C) proteins treated with different doses of Ginkgetin in the PTS assay. (D-E) The melting curves of hcGAS (D) and mcGAS (E) proteins treated with different doses of Ginkgetin in the PTS assay. (F-I) The kinetic binding profiles of Ginkgetin with $hSTING^{REF}$ (F), $hSTING^{Q}$ (G), $hSTING^{N154S}$ (H), and $hSTING^{V155M}$ (I) proteins analyzed by SPR assay. The K_D values were determined using a 1:1 kinetics binding model.

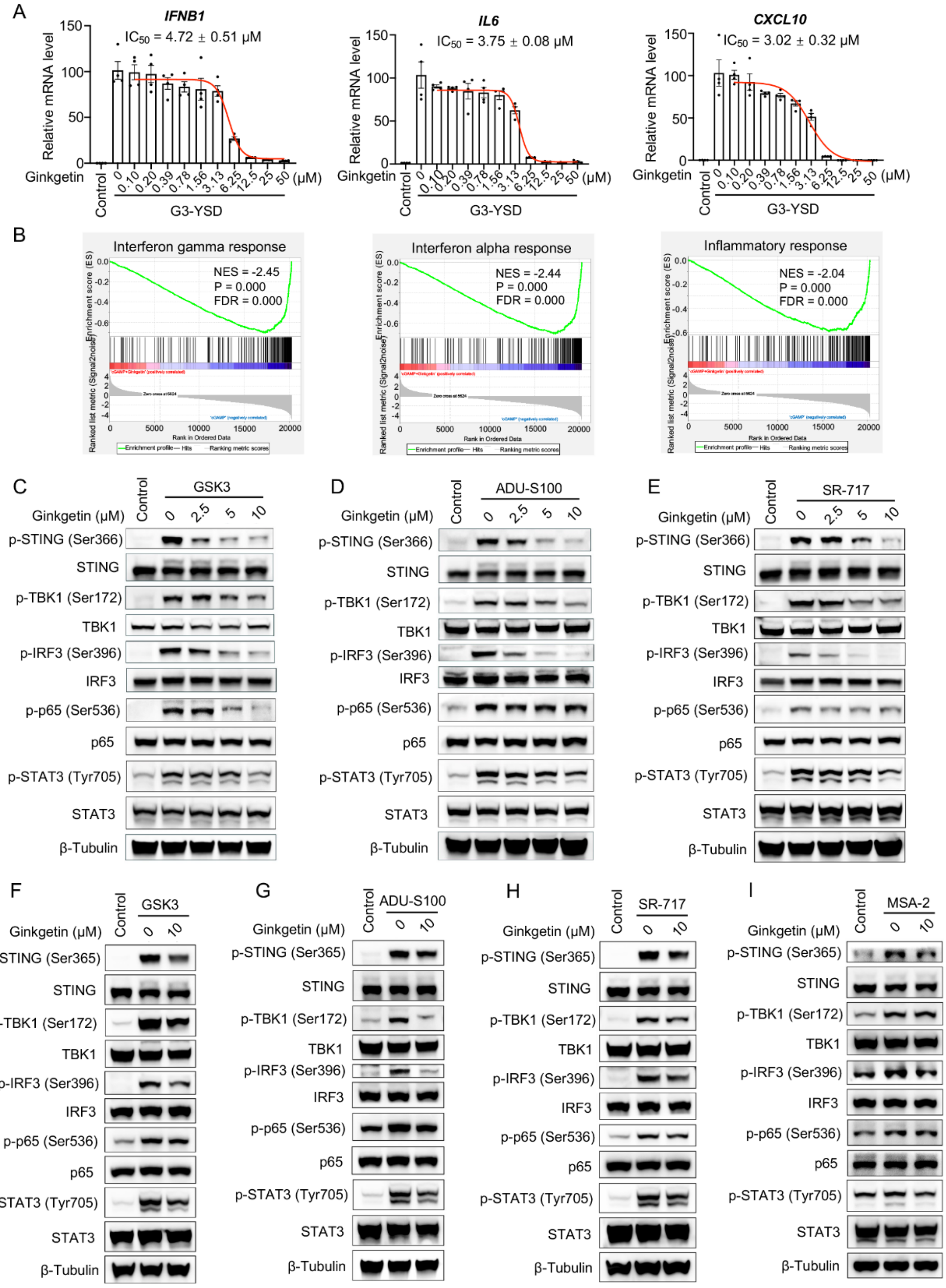

Figure S5. Ginkgetin inhibited STING activation and signal transduction. (A) THP-1 mφs were co-treated with Ginkgetin and G3-YSD for 6 h, and the mRNA expression level of *IFNB1*, *IL6*, and *CXCl10* were measured by RT-qPCR. (B) Transcriptomic analysis was performed on THP-1 mφs treated with indicated compounds for 6 h. The differential genes between the cGAMP group and the Ginkgetin and cGAMP co-treated group were enriched in “Interferon gamma response”, “Interferon alpha response”, and “Inflammatory response”.

(C-E) Western blot analysis of the phosphorylation levels of key proteins in the cGAS-STING pathway in THP-1 mφs co-treated with Ginkgetin and GSK3 (C) or ADU-S100 (D) or SR-717 (E) for 2 h. (F-I) Western blot analysis of the phosphorylation levels of key proteins in the cGAS-STING pathway in Raw 264.7 cells co-treated with Ginkgetin and GSK3 (F) or ADU-S100 (G) or SR-717 (H) or MSA-2 (I) for 2 h.
